# Advances in single-cell long-read sequencing technologies

**DOI:** 10.1093/nargab/lqae047

**Published:** 2024-05-20

**Authors:** Pallavi Gupta, Hannah O’Neill, Ernst J Wolvetang, Aniruddha Chatterjee, Ishaan Gupta

**Affiliations:** University of Queensland – IIT Delhi Research Academy, Hauz Khas, New Delhi 110016, India; Australian Institute of Bioengineering and Nanotechnology (AIBN), The University of Queensland, St Lucia, QLD 4072, Australia; Department of Biochemical Engineering and Biotechnology, Indian Institute of Technology Delhi, Hauz Khas, New Delhi 110016, India; Department of Pathology, Dunedin School of Medicine, University of Otago, 58 Hanover Street, Dunedin 9054, New Zealand; Australian Institute of Bioengineering and Nanotechnology (AIBN), The University of Queensland, St Lucia, QLD 4072, Australia; Department of Pathology, Dunedin School of Medicine, University of Otago, 58 Hanover Street, Dunedin 9054, New Zealand; Department of Biochemical Engineering and Biotechnology, Indian Institute of Technology Delhi, Hauz Khas, New Delhi 110016, India

## Abstract

With an increase in accuracy and throughput of long-read sequencing technologies, they are rapidly being assimilated into the single-cell sequencing pipelines. For transcriptome sequencing, these techniques provide RNA isoform-level information in addition to the gene expression profiles. Long-read sequencing technologies not only help in uncovering complex patterns of cell-type specific splicing, but also offer unprecedented insights into the origin of cellular complexity and thus potentially new avenues for drug development. Additionally, single-cell long-read DNA sequencing enables high-quality assemblies, structural variant detection, haplotype phasing, resolving high-complexity regions, and characterization of epigenetic modifications. Given that significant progress has primarily occurred in single-cell RNA isoform sequencing (scRiso-seq), this review will delve into these advancements in depth and highlight the practical considerations and operational challenges, particularly pertaining to downstream analysis. We also aim to offer a concise introduction to complementary technologies for single-cell sequencing of the genome, epigenome and epitranscriptome. We conclude by identifying certain key areas of innovation that may drive these technologies further and foster more widespread application in biomedical science.

## Introduction

Sequencing the genome, transcriptome and epigenome provide complementary information about a cell's nucleic acids, including their abundance, intracellular distribution and chemical state. Conventional DNA sequencing assumes that all the cells, taken as a bulk sample, contain no underlying mutation (i.e. their genomes are identical). However, cells may harbor single nucleotide variants (SNVs), structural variants (SVs), and copy number variants (CNVs) and carry variable epigenetic signatures. Similarly, traditional bulk RNA sequencing also involves pooling RNA from thousands of cells for sequencing; yielding averaged transcriptome information that ignores variable cell states such as cell cycle phases (1). Furthermore, research into epigenetic alterations has historically been done in bulk cell populations. However, no two cells of an individual are identical in their DNA, RNA, or epigenetic profiles across tissues (2), developmental time points, and disease status (3,4). This necessitates a deeper look into individual cells to understand cellular variation in different contexts. The emergence of single-cell genomic, transcriptomic, and epigenomic sequencing technologies over the years allows for unprecedented resolution of omics profiles at the single-cell level (5).

Single-cell resolution is achieved by attaching short nucleotide tags called cell barcodes (CBs) to the molecules of interest. All such molecules within a cell, typically DNA or RNA, will contain identical barcodes. These allow *in-silico* demultiplexing of sequenced reads to assign them to individual cells. Single-cell sequencing protocols involve PCR amplification to increase the available starting material, which can introduce bias when quantifying the reads. This is especially a concern when determining gene expression or identifying CNVs. To circumvent this issue, unique molecular identifiers (UMIs) are attached to molecules prior to amplification, implying sequenced reads with the same UMI can be removed for a more accurate quantification (6). While UMIs ensure the removal of PCR-generated chimeric fragments that contain more than one UMI, they also allow consensus consequence generation as well as variant calling using all the reads with the same UMI (7). Currently, most of the single-cell sequencing is performed on short-read next-generation sequencing (NGS) platforms which offer cost-effective and high accuracy outputs, which is essential for reliable demultiplexing of cellular barcodes.

Upon performing single-cell RNA sequencing (scRNA-seq), gene expression profiles of individual cells are generally used to group them into clusters that correspond to cell types and/or cell states (8). In addition to differences at the level of gene expression, cells also exhibit alternative splicing to produce different transcript isoforms from the same gene. These include alternative transcription start or termination sites (TSS and TTS), differential exon usage, differential transcript usage, alternative 3′ and/or 5′ splice sites, and intron retention (IR). However, the current scRNA-seq technologies elicit a heavy 3′/5′ bias because only one end of the transcript, generally up to 150 nucleotides, is sequenced (dependent on the library preparation protocol). This prevents accurate determination of true chimeric transcripts generated from chromosomal rearrangements - such as immunoglobulins (Igs) and chimeric RNAs produced in cancers (9,10). Moreover, some scRNA-seq library preparation methods (discussed further in the next section) are dependent on the completeness of 3′ UTR annotation to calculate gene counts. For species where the annotation is incomplete, a significant proportion of reads that map to such loci might be discarded (11).

Genome and epigenome sequencing techniques such as single-cell whole-genome amplification and sequencing (scWGA, scWGS), single-cell assay for transposase-accessible chromatin using sequencing (scATAC-seq), single-cell whole-genome bisulfite sequencing (scWGBS) and single-cell reduced-representation bisulfite sequencing (scRRBS-seq) are constrained by the available starting material that is one diploid DNA molecule per cell. This limitation is further exacerbated in single-cell epigenome profiling, where harsh bisulfite conversion leads to significant loss of DNA (12). Moreover, the short reads obtained have a high mapping uncertainty in repetitive regions, one of the key regions examined in DNA methylation sequencing (13). These may also yield incomplete information when complex structural variations, i.e. a combination of SVs, are prevalent in the genomes (14).

**Figure 1.**
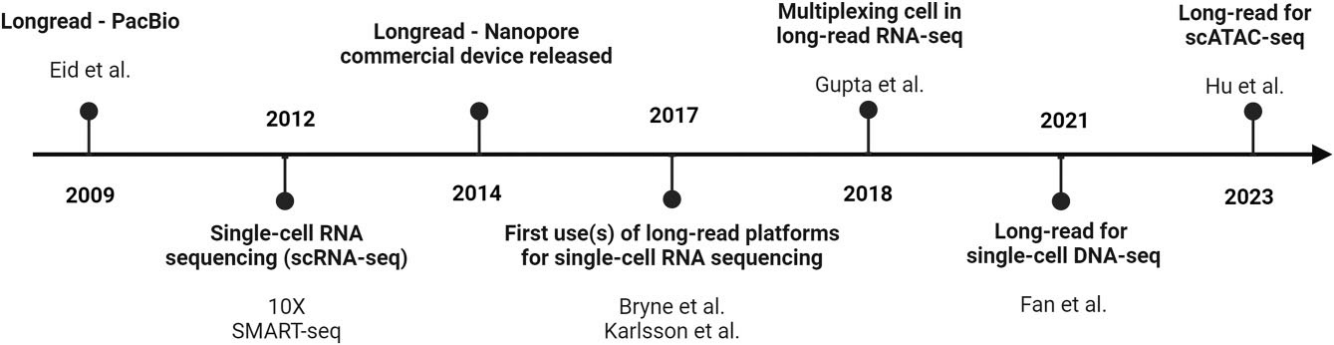
Timeline of technological advancements in single-cell long-read sequencing of the genome, transcriptome and epigenome.

While these technologies have enhanced our understanding of the genome, epigenome, and transcriptome of individual cells, the information is incomplete at many levels. These capability gaps have driven innovations in both library preparation protocols and sequencing technologies that can more accurately identify genomic variants, determine methylation profiles at repetitive elements, capture known as well as novel full-length (FL) isoforms, and determine the extent of alternative splicing at a single-cell level. This has been made possible through third-generation long-read sequencing (TGS) platforms that ideally capture the FL molecules. Figure 1 shows the key advancements of long-read sequencing of various -omes at a single-cell level.

As most of these advances have been made in the field of single-cell RNA isoform profiling, the current review will discuss these in detail while providing a brief overview of the complementary technologies for genome, epigenome, and epitranscriptome sequencing at a single-cell level. We discuss how the long-read sequencing technologies were integrated into existing scRNA-seq pipelines and the challenges pertaining to library preparation, sequencing, and downstream data analysis that these involve.

## Advances in single-cell full-length RNA capture

The library preparation protocols currently used for scRNA-seq are either plate-based, well-based, or droplet-based, depending on the cell separation method (15). Plate-based methods rely on physically isolating cells into 96- or 384-well plates and have low scalability. Microwell-based techniques have higher throughput, but the procedure is lengthy and involves long hands-on time, which can potentially introduce variability due to human error. Conversely, encapsulating cells into individual droplets containing all the required reagents, including barcoded beads, allows parallel sequencing of thousands of cells but demands equal capture rates for all cell types examined in a sample. The emerging combinatorial indexing methods such as sci-RNA-seq and SPLiT-seq, commercialized as SCALE Biosciences and Parse Biosciences respectively, alleviate the requirement of microfluidic devices while ensuring high barcoding sensitivity and throughput (16–18).

The 10X Genomics Chromium assay is a droplet-based library preparation protocol that can then be sequenced on the Illumina platform (19). This method uses a polydT oligo to capture the polyA tail of mRNAs; therefore, only polyadenylated transcripts are captured. Consequently, this method has a lower RNA capture efficiency, more dropout events, and increased technological noise for lowly expressed RNAs. Paired-end sequencing is performed for these samples, where one of the read pairs provides the sequence of the CB and UMI while the other pair captures the transcript (3′ or 5′ end depending on the chemistry used). This technique effectively examines cells from a heterogeneous sample and enables cell clustering based on their gene expression profiles and is widely used. However, to inspect variations in transcript isoforms across cells, the entire length of the isoform must be captured.

An alternative technique is Smart-seq, a plate-based scRNA-seq method that uses tagmentation to capture the transcriptome from short reads while retaining cell information through sample indices analogous to CBs (20–22). Though this technique can delineate relative changes in exon usage, TSS, TTS, and splice sites, it cannot accurately reconstruct FL transcripts, especially if novel transcripts are also expected in the cells. The UMIs are only introduced in Smart-seq3, although most of the fragments along the gene, called internal reads, do not contain these tags as they are only retained in the 5′ fragments (22). This leads to UMI-containing reads having a 5′ bias whereas the others show a 3′ bias, which prevents accurate isoform-level quantification (23).

A bulk RNA-seq technique called LoopSeq tags each fragment of the FL molecule with the same UMI, ensuring more accurate transcript assembly even when performing short-read sequencing (24). It holds the potential to be incorporated into single-cell sequencing workflows, but it remains to be adopted. An innovation called scRCAT-seq (single-cell RNA cap and tail sequencing) and its improvement scRCAT-seq2 were developed to capture variation in transcripts at a single-cell level using short-read sequencing but were limited to the TSS and TTS sequencing (25,26). Another technology, called VASA-seq (vast transcriptome analysis of single cells by dA-tailing), alleviates the issue of bias and single-ended sequencing as observed in Smart-seq and Illumina, respectively, by polyadenylating all fragments to be sequenced (27). However, this approach also suffers from inaccurate assembly of transcripts when multiple isoforms from the same gene are produced. The resulting workflow, hereby called single-cell RNA isoform sequencing (scRiso-seq), can elucidate differences across cells at the isoform-level, including capturing novel isoforms. This has been made possible by using one of the prevalent technologies, such as 10X chromium (droplet-based) and Smart-seq3 (plate-based), to prepare barcoded libraries to multiplex cells while using long-read sequencing platforms instead of the conventional short-read sequencing.

## Choice of long-read sequencing platform for single-cell libraries

Availability of long-read sequencing platforms, the read lengths they offer, their throughput, multiplexing options, and error rates are important considerations for single-cell sequencing. PacBio single-molecule real-time (SMRT) system uses the kinetics of DNA polymerase to incorporate the base and move onto the next—pulse width and interpulse duration, respectively (28). It currently provides Sequel, Sequel II, Sequel IIe and Revio systems that may be used with 1M and 8M SMRT Cells that generate up to 500 000 and 2 000 000 HiFi reads, respectively. The newly launched Revio system offers sequencing in up to four SMRT Cells in parallel. ONT uses the fluctuation in electrical currents to identify the k-mer sequence within a fragment (29). For nanopore sequencing, the MinION, GridION and PromethION devices can be used with either MinION, Flongle, or PromethION flow cells. Illumina also recently introduced synthetic long-read sequencing for the human genome (https://sapac.illumina.com/science/technology/next-generation-sequencing/long-read-sequencing.html).

The early chemistries of PacBio and Nanopore technologies suffered from higher error rates as compared to short-read sequencing platforms, preventing SNV identification and splice-site sequence annotation. Concerning scRiso-seq, this posed a unique challenge—if the error is incorporated in the CB or the UMI, the read(s) will not be accurately assigned. While the accuracies of both long-read platforms have increased over the years, strategies to perform error correction include increasing the coverage of reads, i.e. sequence more fragments corresponding to a region as is performed using consensus circular sequencing (CCS) for PacBio, or using corresponding short reads to perform hybrid error correction (also see subsequent sections).

**Figure 2.**
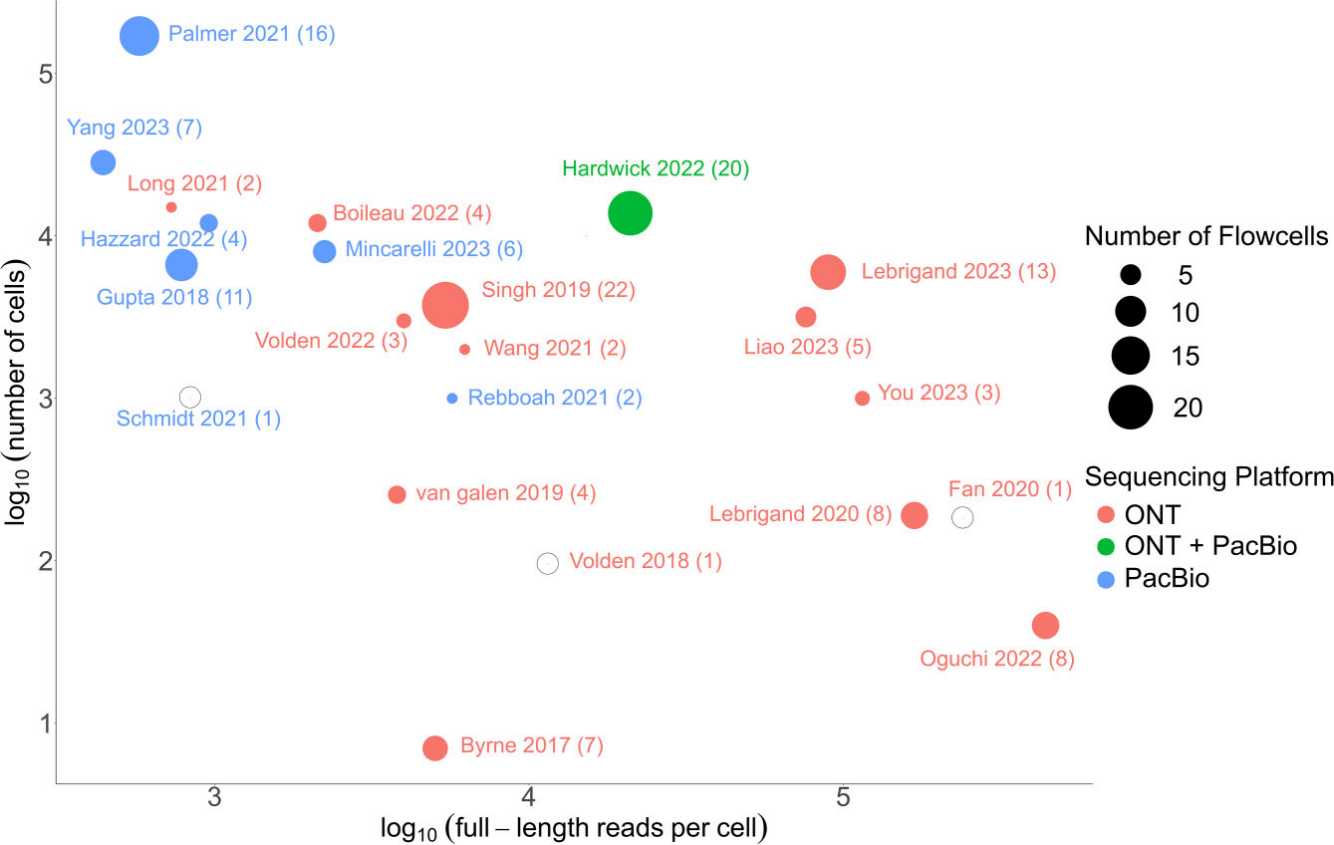
Pictorial representation of studies from Table 1 (24 out of 32) depicting the number of cells sequenced and the average number of FL reads obtained per cell. The number of flow cells from different long-read sequencing technologies used have also been indicated.

Conventionally, for bulk RNA-seq, the library preparation protocol for both long-read sequencing platforms does not include a PCR step. However, to ensure that the few molecules from single cells are captured and sequenced sufficiently, the library prepared for scRiso-seq must be PCR amplified. To assist error-correction in downstream analysis, the single-cell libraries prepared through either of the discussed protocols is split into two - one for long-read and the other for short-read sequencing. For the former, the number of starting cells (tens, hundreds, or thousands depending on the single-cell library preparation method used and its corresponding multiplexing extent), the coverage desired (in terms of FL counts per cell), and the throughput offered by the flow cells from existing platforms affect the choice of the platform used as shown in Table 1. The number of FL reads obtained per cell varies depending on the machine throughput and the input number of cells (Figure 2 provides a graphical representation of this concept).

**Table 1.** A tabular summary of all the peer-reviewed studies till date that perform single-cell long-read isoform sequencing (scRiso-seq)

S. No.	Author year	Total # of cells LR sequenced	Library prep protocol	Sequencing device configuration	FL counts per cell	Total FL counts	Ref	In Figure 2
1	Byrne 2017	7	Smart-seq2	3 cells on 3 R7.3 (2D) + 4 cells on 1 R9.4 (2D) MinION flowcells	35 222 + 93 300	-	(30)	1
2	Karlsson 2017	6	STRT-C1	7 SMRT cells on RSII	NA	NA	(31)	0
3	Gupta 2018	6627	10X 3′ v2 chromium	11 1M SMRT cells on Sequel	-	5.2 × 10^6^	(32)	1
4	Volden 2018	96	Tn5Prime	1 R9.5 (1D) MinION flowcell	-	1 132 707	(33)	1
5	Ranum 2019	12	Smart-seq2	4 R9.4 (1D) MinION flowcells	NA	NA	(34)	0
6	Russel 2019	1614 + 50	10X chromium → PCR enrichment	1 SMRT cell on RSII + 1 SMRT cell on Sequel	NA	NA	(35)	0
7	Singh 2019	3743	10X 3′ v2 chromium	21 R9.4.1 (1D) + 1 R9.5.1 (1D2) MinION flowcells	-	20 346 396	(10)	1
8	van Galen 2019	255	Seq-well WTA	4 R9.4 (1D) / R9.5 (1D2) MinION flowcells	-	0.97 × 10^6^ (3 genes)	(36)	1
9	Fan 2020	183	Smart-seq2	1 PromethION flowcell	-	44 × 10^6^	(37)	1
10	Lebrigand 2020	190 951	10X 3′ v2 chromium	8 PromethION flowcells	1	32 322	(38)	1
11	Wang 2021	2000	10X 3′ v3 chromium	2 R9.4 MinION flowcells	-	12.5 × 10^6^	(39)	1
12	Schmidt 2021*	1000	10X 3′ v3 chromium	1 SMRT cell on Sequel II	-	838 363	(40)	1
13	Palmer 2021	>170 000	10X 3′ v3 chromium	16 SMRT cells on Sequel II	-	98 × 10^6^	(41)	1
14	Long 2021	5000 1000	10X 3′ v2 chromium	2 R9.4.1 MinION flowcells	729 UMI 362 UMI	NA	(42)	1
15	Joglekar 2021	NA	10X 3′ v2 chromium 10X visium	24 SMRT cells on Sequel I + 20 SMRT cells on Sequel II 1 PromethION flowcell			(43)	0
16	Rebboah 2021	1000	Split-seq	2 SMRT cells on Sequel II	-	5 764 421	(44)	1
17	Philpott 2021	1200	Drop-seq	1 PromethION flowcell	NA	NA	(45)	0
18	Tian 2021	2737	10X 3′ v2/v3 chromium + 10X 5′ v1 chromium	4 PromethION flowcells	NA	NA	(46)	0
19	Boileau 2022	12000# spots	10X visium	4 GridION flowcells	-	25.5 × 10^6^	(47)	1
20	Ebrahimi 2022	15375	10X 3′ v3 chromium	NA PromethION flowcells	-	17.5 × 10^6^	(48)	0
21	Hardwick 2022	>13 800	10X 3′ chromium	5 PromethION flowcells + 15 SMRT cells	-	290 × 10^6^	(49)	1
22	Healey 2022	10 000	10X 3′ v3 chromium	1 8M SMRT cell Sequel II	-	152 × 10^6^	(11)	1
23	Volden 2022	3000	10X 3′ chromium	3 MinION + 3 PromethION flowcells	-	12 × 10^6^	(50)	1
24	Oguchi 2022 #	40	SMART-seq v4	8 R9.4 MinION flowcells	-	17.6 × 10^6^	(51)	1
25	Hazzard 2022	12 000	10X 3′ chromium	4 SMRT cells on Sequel II	-	11.5 × 10^6^	(52)	1
26	Liao 2023	3168	Smart-seq3	5 PromethION 48 flowcells	-	241 × 10^6^	(53)	1
27	Shi 2023	2000	10X 3′ v3 chromium	1 8M SMRT cell on Sequel II	-	3.83 × 10^6^	(54)	1
28	Lebrigand 2023	5978	10X visium	13 PromethION	-	535 × 10^6^	(55)	1
29	Mincarelli 2023	>8000	10X 3′ v2 chromium	6 SMRT cells on Sequel/ Sequel II	-	17.9 × 10^6^	(56)	1
30	You 2023	1000	10X 3′ v3 chromium	1 PromethION + 2 GridION flowcells	-	115 × 10^6^	(57)	1
31	Liu 2023	9845	10X 3′ v3 chromium	NA SMRT cells on Sequel II	-	4.96 × 10^6^	(58)	0
32	Yang 2023	28 290	10X 3′ v3 chromium	7 8M SMRT cells on Sequel II	-	12.47 × 10^6^	(59)	1

It lists the number of cells (or spots in spatial transcriptomics) sequenced and the total or per-cell full-length (FL) read counts prior to any processing or quality check. NA: Not available. * indicates the study has not yet been peer-reviewed, # means 60% spot coverage assumed. Note: Since 2020, all ONT sequencing is 1D, therefore not explicitly mentioned.

## Analysis of single-cell long-read RNA-seq data

**Figure 3.**
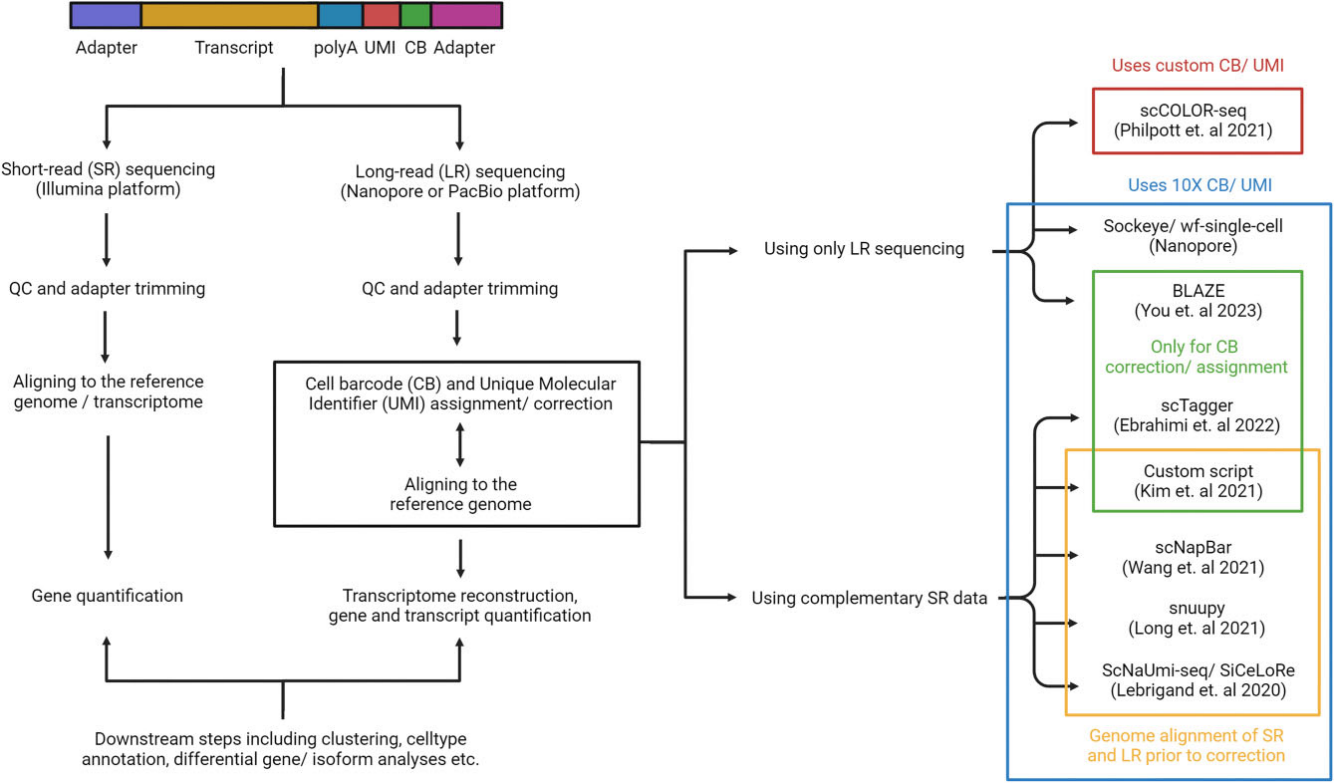
A typical long-read single-cell RNA sequencing (aka scRiso-seq) workflow. The additional step of UMI and CB correction and assignment and the available tools for the same is highlighted.

Once the long-read sequencing run has been performed with or without complementary short-read sequencing, the downstream computational pipeline for an scRiso-seq workflow is outlined in Figure 3. While the initial steps, such as QC and adapter trimming, are performed independently for either technology, data may be merged in the subsequent steps when short-read data is used to correct errors in the long-read set.

### Identifying and correcting CBs and UMIs

Owing to the high error rate of long-read sequencing platforms, one of the essential steps that is different from a standard scRNA-seq workflow is accurately assigning cell barcodes (CBs) and unique molecular identifiers (UMIs) to each read which also harbor these errors. Several bioinformatics tools, combined with various library preparation methods, have been developed to correct and/or assign CBs and UMIs. They are categorized based on whether the algorithm depends on a complementary set of short-read sequences to use as the whitelist for CBs and UMIs.

One of the first publicly-available tools to correctly assign CBs and UMIs using the corresponding short-read data, forming the ScNaUmi-seq (Single-cell Nanopore sequencing with UMIs) workflow, was a Java companion toolkit called sicelore (Single Cell Long Read) (38). Short stretches from the long read between a valid adapter sequence and a threshold number of poly-As contain the CB and UMI. Both the long-read and short-read data are aligned to the genome, and the CBs for each gene or genomic location from both platforms are compared to assign barcodes (see yellow box in Figure 3). Once CBs are correctly assigned to Nanopore reads, UMI sequences for long reads aligning to each gene or genomic location from the same cell are compared to corresponding short-read data. The accuracy of this strategy is over 97% for both CB and UMI assignment and correctly assigned CBs and UMIs for ∼70% of Nanopore reads. As an improvement on SiCeLoRe (38), the authors removed the dependency of the algorithm on the polyA tail to determine the region containing the CB and UMI to develop snuupy (Single Nucleus Utility in Python) (42). In this approach, the unmapped region of the long-read sequences is searched against CB and UMI combinations identified from the short-read sequences. Furthermore, another tool, scNapBar (Single-cell Nanopore Barcode Demultiplexer), also builds on the algorithm of sicelore with an additional aim to reduce the dependency of the algorithm on the depth of short-read sequencing and incomplete genome annotation (39). Especially for low sequencing throughput, instead of using UMI assignment via genome mapping, a Naïve Bayes model is employed to predict the likelihood of the correctness of CB assignment.

Kim et al. also used a similar principle to assign only the CBs at the 5′ end of the transcripts (60). Here too, the soft-clipped or unaligned portion of reads was compared with the corresponding list of CBs from short-read data using cosine similarity scores. The short-read CB with the highest score and the minimum edit distance to the 5′ end is considered the valid match. However, the sample set of CBs in the given study was limited to one amplified gene, and the applicability of this technique to transcriptome-level barcode matching has not been assessed. On the other hand, scTagger is designed to suit both 3′ and 5′ scRNA-seq chemistries to correct CBs (48). It filters out the lowest-frequency barcodes determined from cell ranger (19) and uses the remaining set to assign CBs to long reads. As the library design is known apriori, the regions downstream (or upstream) in forward (or reverse) strands of long reads are compared to the subset of CBs. The CB can be assigned if the computed Levenshtein edit distance is lesser than the user-specific cut-off. Neither of these studies (green box in Figure 3) attempt to correct UMIs; instead perform UMI counting directly.

Two tools developed to identify CBs and/or UMIs when performing only long-read sequencing are Sockeye (https://github.com/nanoporetech/sockeye) and BLAZE (Barcode identification from Long-reads for AnalyZing single-cell gene Expression). Sockeye is used to identify UMIs and CBs in Nanopore reads generated using one of 10X scRNA-seq protocols. However, it does not perform isoform-based single-cell analysis and only returns a gene × cell expression matrix. Benchmarking study also showed that it retains cluster(s) with low UMI count cells and/or non-cell associated barcodes (57). The new implementation of this approach is a Nextflow workflow (https://github.com/epi2me-labs/wf-single-cell), which provides an isoform × cell expression matrix as well. BLAZE uses a three-step approach to identify CBs using only long-read data, making it more conservative than Sockeye (57). Once the putative location of barcodes is identified as immediately downstream of the adapter sequence, those not appearing in the 10X whitelist or with low-quality scores are discarded. Then a quantile-based system is used to retain the high-count CBs.

While the tools mentioned above use the same CBs and UMIs as in the short-read scRNA-seq library preparation protocols (blue box in Figure 3), irrespective of whether short-read sequencing is performed, it is possible to use custom-designed oligos instead when short-read sequencing is not done. This was adopted in scCOLOR-seq - Single-cell Corrected Long-read sequencing (45), where both the oligos are homodimer sequences. This enables the identification and correction of CBs and UMIs using a directional protocol and Levenshtein distances, respectively (61).

### Single-cell long-read RNA-seq analysis pipelines

Once CBs and UMIs have been reliably assigned to FL reads, downstream steps such as aligning to the reference and error correction of the reads, especially of the splice junctions, followed by transcriptome assembly, quantification, and differential transcript analyses can proceed. Most tools for analyzing bulk long-read RNA (or cDNA) sequencing data can be used directly for these purposes. These have been collated in a review elsewhere (62). Analytical toolkits for single-cell data, such as Seurat and Scanpy, can be used to assign cell types or cell states at both gene- and transcript-level (63,64). Tools are also available to visualize (65) interesting transcripts identified through long-read data, including novel fusion transcripts discovered at a single-cell level (66).

Software suites that integrate some of these steps have been designed to aid scRiso-seq processing. Mandalorian was a Python-based pipeline that could perform steps including basic QC, alignment, isoform identification and quantification, and differential isoform usage analysis on ONT 2D reads, which were discontinued in 2017 (30). An updated version could be used for R2C2 reads in combination with the C3POa (Concatemeric Consensus Caller using partial order alignments) consensi-calling tool (33). The FLAMES workflow (Full-Length Analysis of Mutations and Splicing) was designed for CB and UMI assignment in long-read data prior to alignment to the reference genome, using complementary short-read data (46). It subsequently performs downstream steps such as read alignment, transcript assembly, and quantification. The authors reported improved quality of transcript identification over tools such as FLAIR, Stringtie2, and TALON, originally designed for bulk RNA-seq; however, no comparison of CB and UMI assignment accuracy was made. The output from other tools, such as BLAZE (discussed in the previous section), can also be merged with FLAMES to perform only the downstream steps (57). The scISA-tools pipeline was originally developed to prevent the existing scRNA-seq tools from classifying the true full-length non-chimeric (FLNC) reads from HIT-scISOseq as chimeric due to its underlying concatenation principle (54). However, the steps—including reference-guided mapping, UMI and CB error correction (using only long reads), count matrix generation, and cell type annotation—can also be applied to any other scRNA-seq dataset. The scNaST set of tools builds up on scNapBar (39), using a hybrid approach to assign spatial barcodes to long reads, followed by spatial spot deconvolution, spatial gene expression, transcript classification, and differential transcript usage (47). Only recently, two toolkits, scNanoGPS (single-cell Nanopore sequencing analysis of Genotypes and Phenotypes Simultaneously) and Scywalker (not peer-reviewed yet), were developed to assign CBs and UMIs as well as calculate gene and transcript-wise expression profiles without complementary single-cell short reads (67,68). scNanoGPS features CB correction that does not rely on a user-provided barcode whitelist; instead, it is generated within the algorithm. It subsequently identifies duplicate UMIs for reads that map to the same genomic region.

## Single-cell long-read sequencing of alternative -omes

So far, this review has focused on single-cell long-read transcriptome sequencing. However, long-read sequencing of other -omes, such as the genome, epigenome and epitranscriptome at a single-cell level is an emerging area, warrants discussion.

### Single-cell long-read genome sequencing

scWGS or single-cell whole-genome sequencing has allowed small variants in the DNA, such as SNVs, short insertion-deletions (indels), and CNVs, to be identified between cells (69). The existing methods were developed using NGS, where short albeit accurate reads are produced, which are, however, insufficient to detect simple or complex SVs, transposable elements, and extrachromosomal circular DNA (ecDNA) (70). The first report of a single-cell long-read genome sequencing technique was in 2021, termed SMOOTH-seq (single-molecule real-time sequencing of long fragments amplified through transposon insertion) (70). This technology adopted a Tn5 transposition method, previously utilized for scWGS (71), to generate long fragments for third-generation sequencing platforms. Here, instead of two different adapter sequences, the Tn5 transposase with one adapter sequence was used to ensure retrieval of all original DNA fragments. The researchers optimized the reaction conditions to capture and amplify long fragments suitable for TGS platforms efficiently. Briefly, it included the use of Tks Gflex DNA Polymerase for amplification and reduction in the concentration of Tn5 transposase to prevent self-looping. The resulting fragments from each cell are then barcoded and pooled before sequencing.

More recently, a second protocol has emerged that utilizes a droplet-based multiple displacement amplification method (dMDA) (72). Upon the lysis of a single cell, the genomic fragments are encapsulated into droplets such that only one or few DNA molecules are present within each, thereby mitigating the chances of forming inter-molecular chimeras. The small volume and limited reagents in the droplet also reduce overamplification. Once the dMDA occurs, typical library preparation for long-read and short-read sequencing platforms can proceed. While dMDA and similar techniques have been previously described for scWGS or to amplify limited DNA (73,74), this paper is the first to describe its use in long-read sequencing technology.

Pre-processing and genome assembly for either technology proceeds similarly to the corresponding bulk sequencing reads with an extra barcode-based deconvolution step. Downstream applications, such as SNV, CNV or SV identification, can also be performed through the tools available for a typical bulk long-read DNA sequencing analysis. The dMDA method yielded more data per cell, longer read lengths and greater genomic coverage than SMOOTH-seq. The first application of both protocols sequenced a limited number of cell(s) per sequencing run. While SMOOTH-seq had 16 cells per library, producing ∼1 Gb of data per cell, the dMDA method had only one cell per library, producing 20 Gb of data from it. While both the sequencing runs produced HiFi reads, the average read lengths were significantly different—6 kb for the former and 10–12 kb for the latter. The average genomic coverages for the two protocols also differed—19.5% and 40%, respectively. The cost per cell was not stated for dMDA; however, for SMOOTH-seq, the cost of ∼1 Gb per cell was ∼$260. An improved SMOOTH-seq version utilized ONT with the same sequencing depth rather than PacBio, leading to a substantially lower cost of ∼$14 per Gb per cell (75).

### Single-cell long-read epigenome sequencing

Over the years, several single-cell epigenomic methods have been described, exploring DNA methylation (76,77), chromatin accessibility (78), and histone modifications (79). While identifying histone modifications is outside the purview of the technology in discussion, advances in single-cell long-read profiling can boost DNA methylation and chromatin accessibility profiling. However, most epigenomic long-read sequencing techniques have been applied to bulk populations (80–82). PacBio and ONT sequencers rely on the ability of methylated bases to alter the polymerase kinetics and electrical charge pattern, respectively, thus enabling analysis of these DNA modifications (80,83). Currently, the required minimum DNA input for PacBio and ONT is relatively high, with low inputs requiring PCR amplification prior to sequencing (84), while the DNA available from a single cell is ∼6 pg. Unlike single-cell long-read genome sequencing, amplification is not feasible for sequencing the DNA methylome since it would remove the native methylation marks. While bisulfite conversion could still be performed prior to library preparation, it fragments DNA, rendering long-read sequencing futile. Alternative enzymatic reactions for the detection of 5mC and 5hmC using long reads, such as those employed in LR-EM-seq, have been shown to alleviate this shortcoming; however, it is yet to be used for single cells (82). Hence, though there are no publications on single-cell long-read sequencing of DNA methylation to our knowledge yet, as long-read sequencing technologies progress and the technical processes are streamlined, the input requirements may be lowered, making it a more feasible option for single-cell methylome studies.

Only one single-cell long-read epigenomic technique has been described, a single-cell assay for transposase-accessible chromatin on the Nanopore sequencing platform (scNanoATAC-seq) (85). A combination of the plate-based scATAC-seq method (86) with the Tn5 transposition technique was adapted to produce longer fragments for TGS. This allows for the simultaneous analysis of chromatin accessibility and genetic variants such as SVs, SNVs and CNVs within a single cell. This also enables the identification of allele-specific and co-accessible neighboring peaks through haplotype phasing, which is impossible through short-read sequencing. While both SMOOTH-seq and scNanoATAC use Tn5 transposition methods, scNanoATAC-seq has slightly shorter fragments (median 4–5 kb), focusing more on chromatin-accessible regions (85). Up to 960 cells could be multiplexed for a single sequencing run of scNanoATAC-seq on one PromethION flow cell, which reduced the cost to $2.50 per cell. The pre-processing of scNanoATAC-seq reads follows the same steps as bulk ONT. For downstream analyses, the ArchR pipeline (87), originally developed for short-read scATAC-seq, was modified such that only the ends of the long reads were extracted for chromatin accessibility signal analysis.

### Single-cell long-read epitranscriptome sequencing

Similar to epigenomics, epitranscriptomics focuses on the study of functionally relevant RNA modifications that do not alter the underlying ribonucleotide sequence, such as *N*^6^-methyladenosine (m^6^A), 5-methylcytidine, amongst others. These modifications can affect RNA folding, stability, and nuclear export and regulate interaction with other cellular molecules (88). Moreover, different positions of these epitranscriptomic marks regulate transcription differently (89). The ONT sequencing platform can now simultaneously identify isoforms as well as modifications to transcripts, unraveling the multi-layered regulation surrounding the transcriptome. However, like genomic and epigenomic long-read sequencing, publications have focused on bulk populations of cells for epitranscriptome long-read sequencing (90). Theoretically PacBio's kinetics-based sequencing has the capacity to detect RNA base modifications, but the reliable extraction of this information and sequencing of native RNA remains challenging tasks at present (91,92). As with single-cell long-read DNA methylation sequencing, it is not yet possible to carry this out in single cells as the modifications to the RNA are removed during PCR amplification. As the input requirements continue to be lowered for these TGS platforms, this is likely possible in future studies.

## Specific applications of single-cell long-read sequencing

The underlying heterogeneity of ribonucleic acids in nuclear (49), cytoplasmic fractions (51), or total cells (44) can be elucidated through single-cell RNA isoform sequencing. This includes determining the proportion and potential role of compartment-specific transcripts. Some single-cell long-read sequencing strategies aim to enrich target sequences by designing appropriate primers (36) or probe hybridization as used in RAGE-seq (10) and RaCH-seq (93). These methods lead to sequencing cost reduction while investigating informative genes and full-length transcripts. Though scRNA-seq is extensively used to calculate RNA velocity, allowing pseudotime and trajectory analysis, the inability to accurately determine spliced versus unspliced transcripts necessitates several underlying assumptions that can potentially be alleviated by scRiso-seq (62,94). Different developmental stages of the blood parasites, such as *Plasmodium vivax*, have been delineated using FL transcripts from individual cells (52). While the heterogeneity of cells within and across cancer patients can be compared well using this technology, the subpopulations can also be classified based on mutational signatures identified using single-molecule sequencing of transcripts (36). In addition to applying long-read sequencing technologies at a single-cell level, these can also be combined with spatial transcriptomics libraries such as those from 10X visium to detect changes in patterns such as isoform switching across spatial regions (43,47,55). SMOOTH-seq has also demonstrated its effectiveness in detecting structural variants, including duplication events longer than 5 kb, in single cells in a cancer context (70). The group found an enrichment of such events near the telomeres in both colorectal cancer patient samples and K562 cells. This discovery is significant as genomic structural variations are known to drive malignant phenotypes (95,96).

While most of the studies discussed in this review have employed one of the two prevalent technologies, namely 10X chromium or Smart-seq (or its improvements), for library preparation, some authors have also altered these protocols to use in-house tagging (45) and amplification methods instead. Examples include a modification developed for PacBio sequencing called HIT-scISOseq which involves the use of biotinylated primers and concatenation of cDNA inserts to reduce TSO (template switching oligos) artifacts and improve consensus accuracy, respectively (54). Strategies such as SCAN-seq and SCAN-seq2 use identical or different ONT-compatible 24-nucleotide barcodes on 3′ and 5′ ends to tag RNA molecules of each cell to be sequenced (37,53). Another scheme called Rolling Circle Amplification to Concatemeric Consensus (R2C2) circularizes a single-cell library which is then amplified and debranched before sequencing on a Nanopore flow cell (33,50). Furthermore, the improved SPLiT-seq protocol is now also being used in conjunction with long-read sequencing platforms for whole transcriptome sequencing of single cells (44).

## Challenges and future directions

Long-read genome, transcriptome, and epigenome sequencing at a single-cell scale hold immense potential to elucidate individual cell function, regulation, and cellular heterogeneity. However, there are three major challenges that the field currently faces, namely a lack of streamlined library preparation protocols for long-read DNA sequencing of single cells, failure to detect rarer isoforms and cell populations, and the absence of comparative analysis of the existing wet-lab techniques and data analysis pipelines across all omics data. As is evident from the proportion within this review, the field of scRiso-seq has expanded tremendously over the past years, while the exploration of genome, epigenome, and epitranscriptome is either limited or absent. Less than a handful of technologies exist that have not been compared across similar samples. The degree of multiplexing of cells is also limited both at the library preparation step (no droplet-based protocol) and for sequencing (small number of cells per flowcell). Unlike scRNA-seq and scRiso-seq, UMIs are not incorporated into single-cell short-read or long-read DNA sequencing workflows. Although this does not affect SNV identification, it prevents accurate quantification of CNVs. Furthermore, careful consideration must be taken for the genome assembly algorithm(s) used as it can significantly impact the continuity of the assembly (75). Moreover, the sequencing depth in the case of single-cell accessibility profiling (currently using CCS) must be chosen as it offers a trade-off between a greater number of fragments and longer reads, which give stronger epigenetic signals vs. allowing the detection of genomic features, respectively (85).

While capturing every isoform in a cell is of pivotal importance, this largely hinges on the abundances of isoforms themselves, implying that those with low abundance may go undetected entirely. This notion extends to rarer cell types, which are represented by a fewer number of cells and consequently remain uncharacterized or dismissed as spurious signals in single-cell sequencing data. This problem may potentially be alleviated by either increasing the depth of sequencing or sequencing a greater number of cells. Enrichment of certain cells and genes (or isoforms) may also be a potential solution, although it requires prior knowledge about the intended target(s) and this cannot be applied if novel cell types and isoforms are desired in discovery studies.

The third challenge is the unavailability of comparative benchmarking of the available bioinformatics tools and pipelines, especially for CB and UMI assignments in scRiso-seq. Testing these on both simulated and real datasets is necessary for researchers to make an informed decision for their use (97). As the error rates of long-read sequencing platforms is decreasing, such a comparison would enable the users to choose whether to skip corresponding short-read data, without compromising on accuracy, thereby saving both time and money. This demands the development of read simulators that provide the ground truth, which needs improvement (57). Moreover, complications such as chimeric and truncated reads, as well as the high error rates of sequencing, continue to prevent the full utilization of scRiso-seq at single-cell and spatial levels (38,55). Differential gene expression analysis on single-cell data (short-read) poses significant challenges owing to low expression and variability, which requires judicious selection of the best-suited method (98). Such recommendations for method selection must be expanded to long-read single-cell data. Therefore, newer and better library preparation protocols and bioinformatics data analysis pipelines must be developed in the future to address the existing knowledge and capability gaps. These would help advance the field of long-read sequencing of various -omes at a single-cell scale and lead to more widespread deployment of the technologies.

## Data Availability

No new data were generated or analysed in support of this research.
